# Current Final Year Medical Students’ Response to the Varying Attitudes towards Small Group Tutorial Classes Used in Medical Schools in India

**DOI:** 10.30476/JAMP.2021.92492.1491

**Published:** 2022-01

**Authors:** SIMAL ASHER, MOHAMMED BILAL KHAN, KHADIJA HAMID, KIRANDEEP SAHAJPAL, FOZIA KHAN, JUNAYD QADEER

**Affiliations:** 1 Manchester Medical School, Faculty of Biology, Medicine and Health, University of Manchester, Manchester, UK

## Dear Editor

With great interest, we read the article by Shivananda, et al. on the differing perspectives of medical students to small group tutorial classes in India ( 1 ), and we thank the authors for conducting this study. Allowing reflections with our own experiences as final year Medical Students at the University of Manchester, we compared and contrasted these views, and wanted to share our opinions. 

The study does well in establishing that it would be in the best interest of student education within India to increase the availability of smaller group-based teaching within medical schools, evolving from the more traditional didactic learning methods. To yield an improved satisfaction rate in small group teaching, we encourage the authors to integrate the flipped classroom (FC) method, incorporated in medical schools within the United Kingdom, whereby students are given a baseline set of information and resources at the beginning of the week (e.g. e-books, formative quizzes), to get prepared for smaller focused group sessions ( 2 ). These clinical case-based activities include discussion-based workshops, which hone in on specific aspects of the content (biopsychosocial, clinical knowledge, basic sciences, etc.). Hew, et al. conducted a meta-analysis, observing student perception on the FC method; it demonstrated drastic increases in understanding, as well as the application of underlying physiology to tackle questions related to clinical practice, with 71% preferring this format to traditional-based learning ( 3 ). Further investigations into student perception between lecture-based and FC learning methods would highlight any significance.

Additionally, the article abstract states it uses the method of convenience sampling to collect data from the students. Upon significant evaluation, it is evident that use of this method can lead to inaccuracies ( 4 ). Convenience sampling is based on participant availability and willingness to partake5 -this can lead to bias in the results for reasons such as, why some students participated in the study and others did not. This means the results may not be representative and thus, the inability of not being able to generalise research findings ( 5 , 6 ) is one of the reasons why the use of this method is discouraged. One suggestion to reduce the high-level bias of convenience sampling could be the use of random sampling as a data collection method. The use of a sampling frame, random selection of potential participants and equal probability of participant selection allows random sampling to produce a more statistically balanced selection of the population ( 4 ). This results in less biased data and more reliable research conclusions.  

The study has collated data on gender and year groups for students, and has stated that there is no significant difference regarding these variables. Nonetheless, they fail to provide quantifiable evidence within the study to support this claim, while not establishing its relevance. Atlasi, et al. observed how learning styles vary in Iranian medical schools between genders, specifically in anatomy courses, and found that 60% of females within the study preferred smaller group working, stating a leniency towards learning in collaborative environments, and better social interaction compared to their male counterparts ( 7 ). Had the authors provided more information as to why this was evident in India, it may prove key in understanding any discrepancies between students in this cohort compared to others using these variables. 

Overall, the article succeeds in establishing the contrasting attitudes to small group tutorial classes in India by medical students. However, conducting further research into the matter and using more reliable and applicable data collection methods may yield stronger evidence to support the claims made by the authors, and develop better techniques in delivering teaching that is well received by students.

## Conflict of Interest:

None Declared.
